# Effects of initiation and titration of a single pre-prandial dose of insulin glulisine while continuing titrated insulin glargine in type 2 diabetes: a 6-month ‘proof-of-concept’ study

**DOI:** 10.1111/j.1463-1326.2011.01459.x

**Published:** 2011-11

**Authors:** D R Owens, S D Luzio, C Sert-Langeron, M C Riddle

**Affiliations:** 1 Diabetes Research Unit, Cardiff University, University Hospital LlandoughPenarth, UK; 2 sanofi-aventisParis, France; 3 Oregon Health and Science UniversityPortland, OR, USA

**Keywords:** basal insulin, basal-plus, rapid-acting insulin, type 2 diabetes mellitus

## Abstract

**Aim:**

Stepwise intensification of insulin treatment to match the progressive decline of endogenous insulin secretion has been shown to be an effective management strategy in type 2 diabetes mellitus (T2DM). The efficacy of initiating and titrating a single bolus dose of insulin glulisine to baseline insulin glargine plus oral hypoglycaemic agents (OHAs) was investigated.

**Methods:**

This was a 6-month, parallel-group, randomized, open-label, Phase IV study conducted in the US, UK and Russia. People with T2DM (HbA_1c_ 7.5–9.5%) using any basal insulin underwent a 3-month run-in period on insulin glargine titrated to optimize fasting blood glucose (BG) control. Those with HbA_1c_ >7.0% were randomized to either continue prior therapy (n = 57) or to add a single dose of insulin glulisine (n = 49) immediately prior to the main meal for a further 3 months. Two different titration algorithms were employed for the bolus dose, targeting 2-h postprandial BG ≤135 mg/dL (≤7.5 mmol/l; Russia and UK) or pre-meal/bedtime BG 100–120 mg/dl (5.5–6.7 mmol/l; US).

**Results:**

HbA_1c_ and fasting plasma glucose levels decreased during the run-in period. In the 3 months after randomization, more participants in the basal-plus-bolus group reached HbA_1c_ <7.0% than the basal-only control group (22.4 vs. 8.8%; p < 0.05), with significantly greater reduction of HbA_1c_ (−0.37 vs. −0.11%; p = 0.0290). Rates of hypoglycaemia and mean weight change were comparable between the treatment groups.

**Conclusions:**

In people with T2DM inadequately controlled on basal insulin plus OHAs, adding a single injection of insulin glulisine prior to the main meal significantly improves glucose control without undesired side effects.

## Introduction

Over time, the management of type 2 diabetes mellitus (T2DM) requires progressive adjustment of therapy to achieve and maintain adequate glycaemic control in an attempt to reduce the risk of vascular complications. The American Diabetes Association (ADA)/European Association for the Study of Diabetes (EASD) consensus statement suggests beginning pharmacotherapy with metformin or, in cases of metformin intolerance, with either a sulphonylurea or a thiazolidinedione, as needed [1]. If, subsequently, individual or combinations of oral hypoglycaemic agents (OHAs) are no longer adequate, introducing insulin therapy in the form of an ‘intermediate-acting’ or ‘long-acting’ (basal) insulin is amongst the next options. The Treat-to-Target Trial showed that initiation and systematic titration of insulin glargine using a simple algorithm enables a majority of individuals to achieve an HbA_1c_ level <7% with low rates of hypoglycaemia [2].

The next therapeutic step has been much debated, the leading options being (i) cessation of basal insulin and substitution with twice-daily injections of premixed (rapid-acting plus longer-acting) insulin preparations, or (ii) progression to a basal–bolus insulin regimen by continuing basal insulin and adding injections of rapid-acting insulin prior to each meal as required [1]. Recognized limitations of the first option include limited flexibility in insulin dose adjustment resulting in the need for relatively strict lifestyle changes, more frequent hypoglycaemia, and greater weight gain [3,4]. The second option is limited by the inconvenience of more frequent self-monitoring of blood glucose (BG) to guide dose titration and the potential need for multiple preprandial (three to four) daily injections. A third alternative is to continue basal insulin and prior OHA therapy and add a single prandial insulin injection prior to an individual's main meal [5]. Evidence for the efficacy and safety of this latter strategy remains relatively limited. Here, we report a multicentre, multinational, ‘proof-of-concept' study of this sequential approach using two different algorithms for introducing and titrating the prandial insulin dose.

## Materials and Methods

### Subjects

Eligible individuals for this study were men or women with T2DM, aged 18–75 years, with a body mass index of 25–45 kg/m^2^, an HbA_1c_ level of 7.5–9.5% and treated with a basal insulin (neutral protamine Hagedorn [NPH] insulin, lente insulin, insulin glargine or insulin detemir) in combination with metformin (≥1 g/day) for more than 3 months. Exclusion criteria included type 1 diabetes, current or anticipated pregnancy; treatment with OHAs alone (including thiazolidinediones) or exenatide, pramlintide or any other insulin preparation (i.e. premixed or short-acting insulins). Individuals with proliferative retinopathy or clinically relevant cardiovascular, hepatic, renal, neurological, other endocrine or major disease were also excluded. Written informed consent was obtained from all participants, and institutional review board/independent ethics committee approval was obtained for each participating study centre.

### Study Design

**Figure 1:**
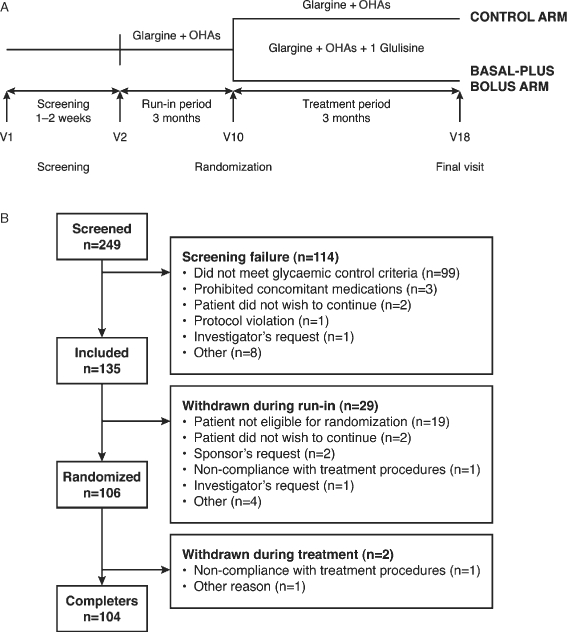
(A) Study design and (B) patient disposition. OHA, oral hypoglycaemic agent; V, visit.

This 6-month, parallel-group, randomized, open-label, Phase IV study was conducted at 12 sites in the UK, 17 in the US and four in Russia. It included an initial 1- to 2-week screening phase followed by a 3-month run-in period and a 3-month randomized treatment period (figure 1A). There were 11 face-to-face visits at weeks −2 (selection), 0 (study entry), 2, 4, 8, 10 (randomization), 12, 14, 16, 20 and 24 (final visit), plus telephone contact at weeks 1, 3, 6, 13, 15, 18 and 22.

During the run-in period, patients were transferred from their prior basal insulin (if other than insulin glargine) to insulin glargine, which was titrated seeking to achieve a fasting BG target of ≤100 mg/dl (≤5.5 mmol/l) using the Treat-to-Target Trial algorithm [2]. At the end of the run-in period, individuals whose HbA_1c_ remained ≥7.0% were randomized (1 : 1), using an interactive voice response telephone system, either to continue their current therapy (basal-only group) or to add one dose of insulin glulisine prior to their main meal (basal-plus-bolus group). Both treatment strategies were continued for a period of three additional months (randomized treatment period).

Titration of the prandial dose of insulin glulisine was performed once a week using one of two different algorithms (Table 1). In the UK and Russia, insulin glulisine dose adjustment was based on the 2-h, postprandial BG with a target of ≤135 mg/dl (≤7.5 mmol/l), whereas in the US the target was a pre-meal BG level of 100–120 mg/dl (5.5–6.7 mmol/l) following the main meal, or at bedtime if the main meal was dinner. The main meal was defined as the meal resulting in the highest postprandial BG value determined from a seven-point daytime BG profile (before and 2 h after each meal, and at bedtime) conducted on three separate days in the week prior to the randomization visit. The timing of insulin glulisine administration was kept constant throughout the 3-month randomized treatment period. Mean daily BG was calculated as the mean of the seven-point BG profiles performed before each visit and variability was characterized by the standard deviations for the mean BG values.

**Table 1:** Insulin glulisine dose titration

**Russia and United Kingdom**	
*Calculation of the initial dose of insulin glulisine for the first injection:*	
Dose of insulin glulisine prior to the main meal = PPBG of the main meal in mmol/l divided by 2	
Treat to target: PPBG ≤135 mg/dl (7.5 mmol/l)	
PPBG ≤135 mg/dL (7.5 mmol/l)	No change
135 mg/dl (7.5 mmol/l) <PPBG ≤153 mg/dl (8.5 mmol/l)	+1 U
153 mg/dl (8.5 mmol/l) <PPBG ≤80 mg/dl (10 mmol/l)	+2 U
PPBG >180 mg/dl (10 mmol/l)	+3 U
At the discretion of the investigator, small decreases of 1 U of the dose of insulin glulisine are permitted in case of hypoglycaemia	
**United States**	
*Calculation of the initial dose of insulin glulisine for the first injection:*	
6 U prior to the main meal. At the same time, the insulin glargine dose was reduced by 6 U and then titrated again the next week	
Treat to target: 100 mg/dl (5.6 mmol/l) <BG*≤120 mg/dl (6.7 mmol/l)	
120 mg/dl (6.7 mmol/l) <BG*≤140 mg/ dl (7.8 mmol/l)	+1 U
140 mg/dl (7.8 mmol/l) <BG*≤180 mg/ dl (10 mmol/l)	+2 U
BG* >180 mg/dl (10 mmol/l)	+2 U
At the discretion of the investigator, small decreases of 1 U of the dose of insulin glulisine are permitted in case of hypoglycaemia	

* Pre-meal or bedtime BG value. FPG, fasting plasma glucose; PPBG, postprandial blood glucose; BG, blood glucose.

The primary outcome was the proportion of patients achieving HbA_1c_< 7.0% at endpoint (with last observation carried forward). Secondary outcomes included changes in HbA_1c_, BG profiles, weight, insulin dose and rates of hypoglycaemia.

Hypoglycaemic episodes were assessed and categorized as asymptomatic, symptomatic, nocturnal symptomatic, severe and severe nocturnal. Asymptomatic hypoglycaemia was an event without clinical symptoms and confirmed by a documented BG level <60 mg/dl (3.3 mmol/l). Symptomatic hypoglycaemia was defined as an event with clinical symptoms consistent with hypoglycaemia, with or without a confirmatory BG measurement, and associated with prompt recovery after oral carbohydrate administration. Severe hypoglycaemia was defined as an event with symptoms consistent with hypoglycaemia in which the patient required assistance from another person, and the event was either confirmed by a BG <36 mg/dl (2.0 mmol/l) or recovery after oral carbohydrate, intravenous glucose or glucagon administration.

### Statistical Analysis

All primary and secondary outcomes, with the exception of hypoglycaemia and adverse events, were assessed in a modified intent-to-treat (mITT) population, defined as all randomized subjects who received study medication and who had an HbA_1c_ value recorded at enrolment and during the randomized treatment period. Additional analyses for the primary outcome and change in HbA_1c_ were also performed for the per-protocol population (PPP), a subset of the mITT population that excluded patients with a major protocol violation. The incidence and rates of hypoglycaemia and occurrence of adverse events were analysed in the safety population, comprising all treated patients. A chi square (*χ*^2^) test was used to compare the percentage of patients with HbA_1c_ < 7% at endpoint. Analysis of covariance was used for continuous variables, with treatment used as the fixed effect and baseline value as a covariate. Missing data were imputed by carrying the last observation forward.

At least 98 patients were needed to be randomized (49 in each arm) in order to demonstrate with 80% power that the addition of a single injection of insulin glulisine prior to the main meal would bring over 40% of patients inadequately controlled with insulin glargine alone plus OHAs to an HbA_1c_ < 7% compared with 15% of patients continuing insulin glargine only plus OHAs (5% alpha risk, two-sided test). A final sample size of 196 patients was calculated by estimating that ∼50% of patients would probably not be eligible for randomization having achieved an HbA_1c_ level <7.0% during the 3-month run-in period or due to exclusion for other reasons.

## Results

### Participant Disposition

Patient enrolment commenced on 9 July 2006 and follow-up of the last patient was completed on 20 August 2008. Of 249 candidates screened, 135 were deemed eligible and willing to enter the run-in period (figure 1B). Of the 29 participants withdrawn from the study during the run-in period, 19 were because of achievement of an HbA_1c_ level <7% on insulin glargine alone. Thus, 106 patients were randomized, 57 to the basal-only group and 49 to the basal-plus-bolus group (PPP:n = 51and45). Characteristics of the total study population at selection and the mITT population at selection and at randomization are included in Table 2. Characteristics of the randomized participants were comparable with the total population and were balanced between the two treatment groups. One participant from each of the treatment groups withdrew during the randomized treatment period; the reasons for withdrawal recorded were ‘non-compliance with treatment procedures' and ‘site error’.

**Table 2:** Subject characteristics at selection and at randomization

		Intention-to-treat population at selection			Intention-to-treat population at randomization		
			
	Total population at selection	Control	Basal plus bolus	Total	Control	Basal plus bolus	Total
n	135	57	49	106	57	49	106
Males, n (%)	58 (43.0)	22 (38.6)	20 (40.8)	42 (39.6)	22 (38.6)	20 (40.8)	42 (39.6)
Age, years	59.3 ± 8.1	59.3 ± 8.8	60.6 ± 6.7	59.9 ± 7.9	—	—	—
Race, n (%)							
White	112 (83.0)	48 (84.2)	43 (87.8)	91 (85.8)	—	—	—
Black	13 (9.6)	6 (10.5)	3 (6.1)	9 (8.5)	—	—	—
Asian-Oriental	4 (3.0)	1 (1.8)	1 (2.0)	2 (1.9)	—	—	—
Multi-racial	1 (0.7)	—	—	—	—	—	—
Other	5 (3.7)	2 (3.5)	2 (4.1)	4 (3.8)	—	—	—
Weight, kg	92.2 ± 17.1	92.3 ± 16.7	91.4 ± 16.1	91.9 ± 16.3	92.9 ± 17.2	91.5 ± 16.6	92.3 ± 16.8
Body mass index, kg/m^2^	33.1 ± 5.0	33.1 ± 4.2	33.0 ± 5.1	33.1 ± 4.6	33.3 ± 4.4	33.2 ± 5.3	33.3 ± 4.8
HbA_1c_, %	8.5 ± 0.6	8.6 ± 0.6	8.5 ± 0.6	8.5 ± 0.6	8.0 ± 0.7	7.8 ± 0.6	7.9 ± 0.6
7–7.5%, n (%)	—	—	—	—	16 (28.1)	17 (34.7)	33 (31.1)
7.5–8.5%, n (%)	66 (48.9)	23 (40.4)	23 (46.9)	46 (43.4)	27 (47.4)	25 (51.0)	52 (49.1)
8.5–9.5%, n (%)	69 (51.1)	34 (59.6)	26 (53.1)	60 (56.6)	13 (22.8)	7 (14.3)	20 (18.9)
>9.5%, n (%)	—	—	—	—	1 (1.8)	—	1 (0.9)
FPG, mg/dl	143.4 ± 39.9	141.8 ± 38.5	147.2 ± 39.8	144.3 ± 39.0	110.9 ± 22.5	111.9 ± 22.6	111.4 ± 22.5
(mmol/l)	(8.0 ± 2.2)	(7.9 ± 2.1)	(8.2 ± 2.2)	(8.0 ± 2.2)	(6.2 ± 1.2)	(6.2 ± 1.3)	(6.2 ± 1.2)
Diabetes duration, years	11.7 ± 7.4	11.0 ± 7.0	12.1 ± 7.3	11.5 ± 7.1	—	—	—
Duration of OHA therapy, years	9.7 ± 6.5	8.6 ± 4.4	11.0 ± 7.5	9.7 ± 6.1	—	—	—
Duration of insulin therapy, years	2.3 ± 2.6	2.0 ± 2.3	2.5 ± 2.7	2.2 ± 2.5	—	—	—
*Insulin use at selection*							
NPH insulin					—	—	—
n (%)	20 (14.8)	8 (14.0)	8 (16.3)	16 (15.1)			
U	40.6 ± 31.1	36.9 ± 20.3	36.3 ± 40.6	36.6 ± 31.0			
Insulin glargine					—	—	—
n (%)	103 (76.3)	46 (80.7)	37 (75.5)	83 (78.3)			
U	38.6 ± 25.1	38.1 ± 24.4	37.0 ± 25.3	37.6 ± 24.7			
Insulin detemir					—	—	—
n (%)	12 (8.9)	3 (5.3)	4 (8.2)	7 (6.6)			
U	44.8 ± 23.0	50.0 ± 24.3	39.0 ± 15.1	43.7 ± 18.6			
*Insulin use at study entry*							
Insulin glargine, U (U/kg)	—	—	—	—	37.0 ± 22.1	36.5 ± 25.6	36.8 ± 23.7
					(0.40 ± 0.22)	(0.40 ± 0.26)	(0.40 ± 0.24)
*Insulin use at randomization*							
Insulin glargine, U (U/kg)	—	—	—	—	55.2 ± 28.3	52.8 ± 31.3	54.1 ± 29.6
					(0.59 ± 0.26)	(0.57 ± 0.31)	(0.58 ± 0.28)
Insulin glulisine, U (U/kg)	—	—	—	—	—	5.4 ± 1.0	
						(0.06 ± 0.01)	—

Results are n (%) or mean ± standard deviation. FPG, fasting plasma glucose; OHA, oral hypoglycaemic agent.

### Glycaemic Responses to Therapy

Transfer to insulin glargine and subsequent titration of dosage during the run-in period improved the mean HbA_1c_ from 8.5 ± 0.6% at selection to 7.9 ± 0.6% at randomization and fasting plasma glucose (FPG) from 144.3 ± 39.0 to 111.4 ± 22.5 mg/dl (8.0 ± 2.2 to 6.2 ± 1.2 mmol/l), respectively (Table 2). After 3 months of randomized treatment, the reduction in HbA_1c_ from the time of randomization was significantly greater in the basal-plus-bolus group than in the basal-only group (−0.37 vs. −0.11%; p = 0.0290; Table 3). This finding was confirmed in the PPP (−0.40 vs. −0.13%; p = 0.0242). Reductions in mean daily plasma glucose (PG) were also significantly greater in the basal-plus-bolus group (−15.0 vs. −2.1 mg/dl [−0.8 vs. 0.1 mmol/l]; p = 0.011; Table 3). The variability in mean daily PG improved in the basal-plus-bolus group over the course of the randomized treatment period, whilst remaining essentially unchanged in the basal-only group (Table 3).

**Table 3:** Clinical outcomes at study end

Parameter	Control	Basal plus bolus	p value
**n**	57	49	
**HbA**_**1c**_			
<7%, n (%)	5 (8.8)	11 (22.4)	0.0499*
Total (%)	7.8 ± 0.9	7.5 ± 0.6	—
Adjusted change (%) (Endpoint–randomization)	−0.11 ± 0.08	−0.37 ± 0.09	0.0290†
**Daily mean PG, mg/dl (mmol/l)**			
Randomization	167.4 ± 39.4	170.2 ± 27.9	—
	(9.3 ± 2.2)	(9.5 ± 1.6)	
Endpoint	165.8 ± 37.5	154.7 ± 28.6	—
	(9.2 ± 2.1)	(8.6 ± 1.6)	
Adjusted change	−2.1 ± 3.4	−15.0 ± 3.7	0.0109†
(Endpoint–randomization)	(−0.1 ± 0.2)	(−0.8 ± 0.2)	
**Daily variability in PG, mg/dl (mmol/l)**			
Randomization	50.6 ± 26.9	49.1 ± 18.4	—
	(2.8 ± 1.5)	(2.7 ± 1.0)	
Endpoint	50.0 ± 21.7	44.7 ± 21.0	0.043‡
	(2.8 ± 1.2)	(2.5 ± 1.2)	
**Daily insulin dose, U (U/kg)**			
*Insulin glargine*			
Randomization	55.2 ± 28.3	52.8 ± 31.3	—
	(0.59 ± 0.26)	(0.57 ± 0.31)	
Endpoint	62.2 ± 34.9	54.7 ± 34.8	—
	(0.65 ± 0.32)	(0.59 ± 0.35)	
*Insulin glulisine*			
Randomization	—	5.4 ± 1.0	—
		(0.06 ± 0.01)	
Endpoint	—	12.8 ± 6.6	—
		(0.14 ± 0.07)	

Data are n (%), means ± standard deviation, or adjusted means ± standard error for change.

* Chi square test.

† Analysis of covariance (ANCOVA) analysis on change adjusted on randomization value.

‡ ANCOVA analysis on the rank variability at endpoint adjusted on the rank randomization variability. PG, plasma glucose.

The primary endpoint, that is, achievement of an HbA_1c_ level <7% at the end of the randomized treatment period, was reached more frequently by participants in the basal-plus-bolus group than those in the basal-only group (mITT: 22.4 vs. 8.8%; p < 0.05; PPP: 24.4 vs. 7.8%, p = 0.0254) (Table 3). Within the basal-plus-bolus group, 10, 14 and 25 patients injected insulin glulisine either before breakfast, lunch or dinner, respectively. Of these, two (20.0%), three (21.4%) and six (24.0%) reached HbA_1c_ < 7% at endpoint, respectively. The mean HbA_1c_ at endpoint in these three subgroups of the basal-plus-bolus arm was 7.6 ± 0.7, 7.6 ± 0.8 and 7.4 ± 0.5%, respectively, with no difference between the groups. Few patients achieved an HbA_1c_ level <6.5% at the end of randomized treatment in either group (mITT: basal-plus-bolus group n = 2 [4.1%]; basal-only group n = 1 [1.8%].)

The reduction in HbA_1c_ from randomization was greater with the US titration algorithm compared with the version used in the UK and Russia without reaching statistical significance (adjusted mean change from randomization to study end: −0.44 vs. −0.18%; p = 0.0603). In addition, a greater proportion of patients in the mITT achieved target HbA_1c_ < 7.0% with the US titration algorithm versus the algorithm used in the UK and Russian centres, but this difference was also not statistically significant (34.8 vs. 11.5%; p = 0.0516).

### Insulin Doses and Distribution

The mean daily doses of NPH, insulin glargine and insulin detemir used by participants in the mITT population prior to study entry were 36.6, 37.6 and 43.7 units, respectively (Table 2). In both treatment groups, the daily dose of basal insulin glargine increased between the time of inclusion and randomization by 16.3 ± 14.7 and 18.5 ± 14.5 units in the basal-plus-bolus and basal-only groups, respectively. At the end of the run-in period, participants used a mean daily dose of 54.1 units of insulin glargine, with a comparable dosage between the basal-plus-bolus and basal-only groups (Table 3). Between randomization and endpoint, basal insulin doses increased by 2.0 ± 10.2 units in the basal-plus-bolus group and 6.8 ± 11.3 units in the basal-only group (Table 3). Within the basal-plus-bolus group, mean doses of insulin glulisine initially recommended at randomization were 5.3 ± 0.8, 5.6 ± 0.9 and 5.4 ± 1.0 units for participants taking insulin glulisine at breakfast, lunch or dinner, respectively. These increased to 10.9 ± 7.2, 12.2 ± 6.8 and 14.2 ± 6.3 units, respectively, at the last visit.

Mean insulin glargine and insulin glulisine doses at endpoint were numerically higher for patients treated with the US versus the UK/Russia titration algorithm. At study end, insulin glargine doses were 0.64 ± 0.45 U/kg and 0.54 ± 0.22 U/kg with the US and UK/Russia algorithms, respectively. Similarly, insulin glulisine doses at study end were 0.17 ± 0.08 U/kg and 0.11 ± 0.05 U/kg, respectively.

### Seven-Point Glucose Profiles

**Figure 2:**
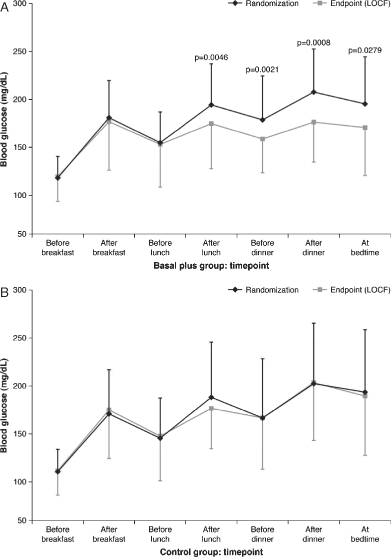
Seven-point self-monitored blood glucose profiles in the bolus (A) and control (B) groups. Blood glucose was monitored before breakfast (fasting), lunch and dinner, 2 h after each meal, and before bedtime. Results are means ± standard deviation. LOCF, last observation carried forward.

**Figure 3:**
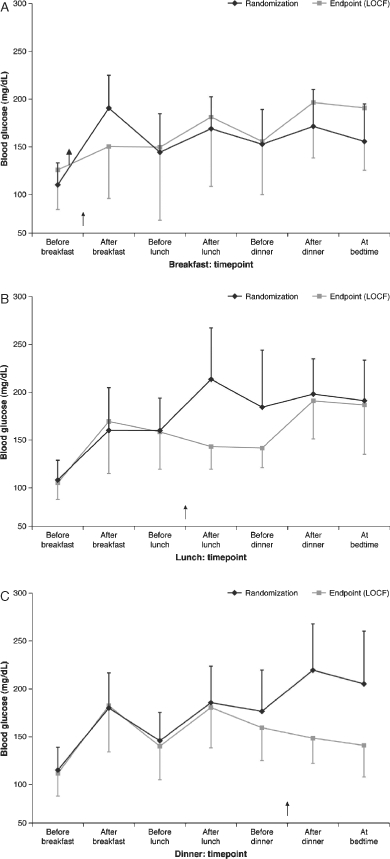
Effect of time of insulin glulisine injection (breakfast, n = 10 [A]; lunch, n = 14 [B]; dinner, n = 25 [C]) on seven-point self-monitored blood glucose profiles. Results are means ± standard deviation. Arrow indicates time of injection. LOCF, last observation carried forward.

Figure 2 shows the PG profiles obtained at randomization and endpoint for the two treatment groups. In the basal-plus-bolus treatment group, most of the reduction in PG levels over the randomized treatment period occurred after lunch (p = 0.0046), before dinner (p = 0.0021), after dinner (p = 0.0008) and before bedtime (p = 0.0279) (figure 2A). There were no significant differences in the profiles PG at randomization and endpoint in the basal-only group (figure 2B). In the three subgroups of participants taking a bolus injection either at breakfast, lunch or dinner, a reduction in the measurement following the post-injection meal was apparent (figure 3A–C), although these differences were not tested for statistical significance because of the small number of subjects in each subgroup.

### Hypoglycaemia

During the run-in period, a total of 57 patients reported hypoglycaemia, including 50 with symptomatic, 26 with asymptomatic and 29 with nocturnal symptomatic hypoglycaemia. Rates of all, symptomatic, asymptomatic and nocturnal hypoglycaemia were 10.5 ± 21.6, 8.5 ± 18.9, 2.0 ± 5.6 and 2.3 ± 5.9 episodes/patient–year, respectively. No episodes of severe hypoglycaemia were reported during the run-in period. The frequency of hypoglycaemia during the 3-month randomized treatment period continued to be low and rates did not differ between the treatment groups (all reported hypoglycaemia [mean ± standard deviation] for control vs. basal plus bolus groups, respectively: 11.1 ± 15.5 vs. 13.4 ± 21.7 events/patient–year, p = 0.960; symptomatic hypoglycaemia: 8.9 ± 15.2 vs. 10.9 ± 18.2, p = 0.544; asymptomatic hypoglycaemia: 2.3 ± 5.2 vs. 2.5 ± 7.7, p = 0.936; nocturnal symptomatic hypoglycaemia: 4.4 ± 10.2 vs. 2.0 ± 4.5, p = 0.307); severe symptomatic hypoglycaemia: 0.2 ± 1.1 vs. 0.0 ± 0.0, p = 0.192; nocturnal severe symptomatic hypoglycaemia: 0.1 ± 1.0 vs. 0.0 ± 0.0; p = 0.364; p values calculated using the Wilcoxon rank sum test).

### Weight Change

Weight change during both the run-in period and the randomized treatment period was small and not statistically significant. Mean bodyweight was 92.1 and 91.5 kg at selection, 92.9 and 91.5 kg at randomization and 92.5 and 92.2 kg at last observation in the control and bolus groups, respectively. There were no statistically significant differences in weight change between the treatment groups.

### Safety and Tolerability

Three subjects experienced a serious adverse event in the run-in period prior to randomization that required hospitalization; they included angina pectoris, food poisoning and hypertensive crisis (n = 1each). During the randomized treatment period, 20 (40.8%) and 28 (49.1%) subjects in the basal-plus-bolus and basal-only control groups reported an adverse event, respectively. Four adverse events during the treatment period were considered to be serious, of which two were in the basal-plus-bolus group (angina pectoris and atrial fibrillation) and two in the basal-only group (scleroderma and tendon disorders). There were no deaths reported during the study.

## Discussion

These results show that the addition of a single dose of insulin glulisine prior to the meal with the greatest postprandial glucose excursion to ongoing basal insulin glargine plus OHAs significantly improved glycaemic control in terms of mean HbA_1c_ levels. Mean daily PG levels, variability in mean daily PG and the diurnal PG profile were also improved with the basal-plus-bolus strategy versus basal only. Improved glycaemic control was achieved without significant increases in the frequency of hypoglycaemia or weight gain. The findings of this study are consistent with those of the Orals Plus Apidra and LANTUS (OPAL) study [5] in which one injection of insulin glulisine added to insulin glargine plus OHAs significantly improved HbA_1c_ levels in people with T2DM, irrespective of whether the prandial insulin was administered at breakfast or at the main meal. The important difference between this and the OPAL study is that the present study had a run-in period to dose titrate insulin glargine prior to the addition of a single dose of insulin glulisine in individuals with inadequate glycaemic control whilst on insulin glargine plus OHAs.

Both fasting hyperglycaemia and increments of glucose after meals contribute to overall glycaemic exposure. After improvement of fasting hyperglycaemia with basal insulin therapy, much of the remaining glycaemic exposure is due to postprandial excursions [6,7]. Therefore, when HbA_1c_ remains higher than 7% after fasting glucose has been normalized or nearly normalized with basal insulin, further improvement requires attention to postprandial hyperglycaemia. The potential benefits of limiting glycaemic excursions may extend to reducing cardiovascular risk from reduced glycaemic variability [8,9]. The ADA/EASD consensus statement recommends that attempts to intensify insulin therapy should be undertaken if HbA_1c_ levels remain above target on a basal insulin-only regimen [1]. However, methods to achieve this treatment intensification have not been fully investigated. In clinical practice, the main approaches are to either add short-acting insulin at each meal (basal–bolus) or switch to twice- or three-times-daily premixed insulin regimens. Both approaches are relatively complex. Individuals and clinicians may experience difficulties in optimizing multiple insulin doses while avoiding hypoglycaemia thereby necessitating frequent self-monitoring of blood glucose. It is likely that the well-documented delay in advancing therapies (clinical inertia) results in part from these complexities [10].

The findings in this proof-of-concept study suggest that a strategy of adding a single prandial insulin injection to basal insulin may offer a more logical initial step for the intensification of insulin therapy in T2DM. The overall reduction in HbA_1c_ was relatively small, but was achieved in just 3 months of treatment, and without accompanying difficulty with hypoglycaemia or weight gain. This method might be most effective for individuals whose HbA_1c_ values are only modestly above the 7.0% target, perhaps in the 7.0–7.5% range. In the present study population, participants had been on insulin for at least 2 years, and their mean HbA_1c_ level was 8.5% at enrolment and 7.9% at randomization, so that relatively few attained the <7.0% goal. However, this first step in introducing prandial insulin therapy may provide a starting point for advancing to full basal–bolus treatment for those needing further intensification given individual clinical requirement based on diabetes duration, age and risk of macrovascular disease [11,12].

As a proof-of-concept study, this study has several limitations. The number of patients enrolled was relatively small, and the relatively short period of use of basal plus prandial therapy probably underestimated the potential glucose lowering that might have resulted had dose-titration and follow up continued beyond 3 months. The clinical results with the two algorithms employed were not clinically or statistically different. There was a trend in favour of the US algorithm, but the numbers of participants studied were too small to permit a conclusion on this point. In addition, assignment of the algorithms depended on country of residence rather than randomization and so any comparison may be open to bias due to country-specific factors. Further studies are necessary to adequately compare these two algorithms.

In conclusion, this study clearly supports the rationale, safety and efficacy of adding a single dose of insulin glulisine to ongoing insulin glargine plus OHAs to improve HbA_1c_ and mean daily plasma BG when HbA_1c_ targets have not been met. This approach may serve as the first logical step from basal insulin plus OHA therapy to a more intensive insulin regimen in the quest to achieve glycaemic targets that are appropriate for clinical needs without requiring a full basal–bolus regimen, with its inherent demands on the individual and their carer.
